# Comment on: Textbook Outcomes Following Open Live Donor Right Hepatectomy and Open Right Hepatic Lobectomy for Cancer in 686 Patients: Re-Defining the Benchmark

**DOI:** 10.1097/AS9.0000000000000244

**Published:** 2023-01-30

**Authors:** Zhi-Peng Liu, Hai-Su Dai, Yi-Shi Yang, Xiao-Yu Che, Zhi-Yu Chen

**Affiliations:** From the Department of Hepatobiliary Surgery, Southwest Hospital, Third Military Medical University (Amy Medical University), Chongqing, China.

We read the article by Dogeas et al^1^ with great interest. This study used a prospective database to compare textbook outcomes (TO) rates between right hepatectomy (RH) for live donor purposes and RH for malignancy. The author found that the TO rates of RH for live donor purposes (92.2%) were significantly higher than that for malignancy (53.7%) and considered that these indicators represented a new benchmark for “real world” TO after open RH. Despite inspiring and thought-provoking, we have the following comments.

First, in our experience, in the real world, regardless of the type of hepatectomy, the most common cause is hepatocellular carcinoma. However, among cancer patients in this study, the most common pathology was colorectal cancer liver metastases, accounting for 58.4% (184 patients). This also means that cirrhosis has a very low proportion of cancer patients, only 5.4% (17 patients). In fact, there may be a higher incidence of cirrhosis in all cancer patients who underwent RH. Although cirrhosis was only confirmed as an independent risk factor associated with a lower TO rate of hepatocellular carcinoma hepatectomy in our previous study,^2^ we still believe that cirrhosis may strongly affect the TO rate in all cases of hepatectomy. Therefore, it is reasonable to wonder whether the TO rate in the tumor patients in this study can be viewed as a new benchmark for the “real world” and whether independent factors associated with not achieving TO can be applied to all RH cancer patients.

Second, this study noted that the live donor RH people had higher TO rates, as they were younger, healthier, and thinner, but logistic regression analysis showed no significant association between body mass index (BMI) and TO rates. The study did not mention the grouping method of BMI in the methods section. If BMI is analyzed as a continuous variable, it is likely to obtain negative results that BMI is not related to TO rates. However, high BMI often means that body obesity, while low BMI means body malnutrition or cachexia. Consequently, appropriate BMI grouping may directly determine the results of the study. We wanted to know how the authors analyzed of the association between BMI and TO rates.

Third, TO rates were compared between RH for live donor purposes and RH for malignancy in this study. However, direct comparisons between the 2 groups may not be statistically appropriate due to the large difference between the 2 groups. Therefore, we believe that some statistical methods, such as propensity score matching or inverse probability of treatment weighting, should be used first to balance the baseline data between the 2 groups, perhaps to make the 2 groups more comparable. Although balancing may not have changed the final results of this study, we still believe that balancing the baselines of the 2 groups and then comparing them can make the results of this study more solid.

Finally, we are still very appreciative of Dogeas et al^1^ for the new benchmark for “real world” TO after RH. We hold the belief that these findings will contribute to the further improvement and development of liver surgery.

## References

[1] Dogeas E Geller DA Tohme S. Textbook outcomes following open live donor right hepatectomy and open right hepatic lobectomy for cancer in 686 patients: re-defining the benchmark [published online ahead of print November 2, 2022]. Ann Surg. doi: 10.1097/SLA.0000000000005749.

[2] Liu ZP Yao LQ Diao YK. Association of preoperative body mass index with surgical textbook outcomes following hepatectomy for hepatocellular carcinoma: a multicenter study of 1206 patients. Ann Surg Oncol. 2022;29:4278–4286. doi: 10.1245/s10434-022-11721-y. 10.1245/s10434-022-11721-y 35419755

